# Multiple Sites of the Cleavage of 21- and 25-Mer Encephalytogenic Oligopeptides Corresponding to Human Myelin Basic Protein (MBP) by Specific Anti-MBP Antibodies from Patients with Systemic Lupus Erythematosus

**DOI:** 10.1371/journal.pone.0051600

**Published:** 2013-03-08

**Authors:** Anna M. Timofeeva, Pavel S. Dmitrenok, Ludmila P. Konenkova, Valentina N. Buneva, Georgy A. Nevinsky

**Affiliations:** 1 Institute of Chemical Biology and Fundamental Medicine, Siberian Division of Russian Academy of Sciences, Novosibirsk, Russia; 2 Pacific Institute of Bioorganic Chemistry, Far East Division, Russian Academy of Sciences, Vladivostok, Russia; 3 Institute of Clinical Immunology, Siberian Division of Russian Medical Academy of Sciences, Novosibirsk, Russia; 4 Novosibirsk State University, Novosibirsk, Russia; University of South Florida College of Medicine, United States of America

## Abstract

IgGs from patients with multiple sclerosis and systemic lupus erythematosus (SLE) purified on MBP-Sepharose in contrast to canonical proteases hydrolyze effectively only myelin basic protein (MBP), but not many other tested proteins. Here we have shown for the first time that anti-MBP SLE IgGs hydrolyze nonspecific tri- and tetrapeptides with an extreme low efficiency and cannot effectively hydrolyze longer 20-mer nonspecific oligopeptides corresponding to antigenic determinants (AGDs) of HIV-1 integrase. At the same time, anti-MBP SLE IgGs efficiently hydrolyze oligopeptides corresponding to AGDs of MBP. All sites of IgG-mediated proteolysis of 21-and 25-mer encephalytogenic oligopeptides corresponding to two known AGDs of MBP were found by a combination of reverse-phase chromatography, TLC, and MALDI spectrometry. Several clustered major, moderate, and minor sites of cleavage were revealed in the case of 21- and 25-mer oligopeptides. The active sites of anti-MBP abzymes are localised on their light chains, while heavy chains are responsible for the affinity of protein substrates. Interactions of intact globular proteins with both light and heavy chains of abzymes provide high affinity to MBP and specificity of this protein hydrolysis. The affinity of anti-MBP abzymes for intact MBP is approximately 1000-fold higher than for the oligopeptides. The data suggest that all oligopeptides interact mainly with the light chains of different monoclonal abzymes of total pool of IgGs, which possesses a lower affinity for substrates, and therefore, depending on the oligopeptide sequences, their hydrolysis may be less specific than globular protein and can occur in several sites.

## Introduction

It is known, that the occurrence of auto-Abs in increased concentration is a distinctive feature of various autoimmune diseases (ADs) (reviewed in [1]–[8]). It was shown that small fractions of auto-Abs can possess different catalytic activities [1]–[8]. Catalytically active artificial antibodies (Abs) or abzymes (Abzs) against transition chemical states of different reactions were studied intensively (reviewed in [1]). Healthy humans and patients with many diseases with insignificant autoimmune reactions usually lack abzymes or develop Abzs with very low catalytic activities, with these activities being often on a borderline of the sensitivity of detection methods (reviewed in [2]–[8]). Natural abzymes hydrolyzing DNA, RNA, polysaccharides, oligopeptides (OPs), and proteins are described from the sera of patients with several autoimmune (systemic lupus erythematosus, Hashimoto's thyroiditis, polyarthritis, multiple sclerosis, asthma, rheumatoid arthritis, etc.) and viral diseases with a pronounced immune system disturbance (viral hepatitis, AIDS, and tick-borne encephalitis) (reviewed in [2]–[10]). Abzymes may play a significant positive and/or negative role in broadening Ab properties, forming specific pathogenic patterns and clinical settings in different autoimmune conditions [1]–[10].

Multiple sclerosis (MS) and systemic lupus erythematosus (SLE) are well known ADs. MS is a chronic demyelinating disease of the central nervous system. Its etiology remains unclear, and the most widely accepted theory of MS pathogenesis assigns the main role in the destruction of myelin to the inflammation related to autoimmune reactions [11]. Several recent findings imply an important role of B cells and auto-Abs against myelin autoantigens including myelin basic protein (MBP) in the pathogenesis of MS [11]–[13].

SLE is a systemic autoimmune polyetiologic diffuse disease characterized by disorganization of conjunctive tissues with the paramount damage to skin and visceral capillaries [14]. The polyetiologic and polysyndromic character of SLE leads to highly variable manifestations of this disease in terms of many biochemical, immunological, and clinical indices. SLE is usually considered to be related to patient's autoimmunization with DNA, since the sera of such patients usually contain DNA and anti-DNA Abs in high concentrations [15]. At the same time, in comparison with healthy donors, an increased concentration of auto-Abs was observed for various antigens (% of patients): DNA (60), cardiolipin (48), thyroglobulin (42), microsomal fraction of thyrocytes (48), and rheumatoid factor (23) [5].

It should be mentioned, that SLE and MS demonstrated some similarity in the development of the same medical, biochemical and immunological indexes. MS is a chronic disease of the central nervous system leading to the manifestation of different nervous and psychiatric disturbances. However, neuropsychiatric involvement occurs in about 50% of SLE patients and carries a poor prognosis (reviewed in [11]). SLE predominantly affects the central nervous system, and within its cerebral complications it has a particular propensity-perhaps more than any other systemic inflammatory disease– to cause psychiatric disorders [11]. Peripheral nervous system involvement is much less common. The distinctive production of diverse auto-Abs seems to be related to defective clearance of apoptotic cells. Antibody-mediated neural cell injury and rheological disturbances represent the two principal suggested mechanisms of tissue injury [11]. Interplay between these processes, underlying genetic factors, their modification by hormones, complicated by a number of secondary factors, may explain the wide spectrum of features encountered in this disease. Some indicators of disease common to SLE and MS were observed [11].

For diagnostics of MS, thirteen Poser's medical indices are often used [16], but clinically definite MS diagnosis is usually based on the tomographic detection of specific plaques in the brain, which appear on late stages of not only this disease, but also in SLE patients. Similarly to SLE, the high-affinity anti-DNA Abs has been recently identified as a major component of the intrathecal IgGs in MS patient's brains and cells of the cerebrospinal fluid [17]. It was recently shown that titers of Abs against human myelin basic protein in SLE patients 4.2-fold higher than in healthy individuals, but 2.1-fold lower than in patients with MS [9]. In addition, abzymes from the sera of patients with SLE and MS possess by the same catalytic activities (see below).

It was shown that SLE IgGs and IgMs effectively hydrolyzed DNA, RNA, and polysaccharides [18]–[22]. Similarly to SLE, homogeneous IgGs from the sera and the cerebrospinal fluid of MS patients were active in the hydrolysis of DNA, RNA, and polysaccharides [23]–[25]. Whereas only 18 and 53% of MS patients contained increased concentrations of Abs to native and denatured DNA, respectively, as compared with healthy donors, DNase abzymes were found in ∼80–90% of MS patients [23]–[24]. Since DNase abzymes of MS patients [4] similarly to SLE patients [26] are cytotoxic and induce apoptosis, they can play an important role in SLE and MS pathogenesis.

It has been recently shown that MBP-hydrolyzing activity is an intrinsic property of IgGs, IgMs, and IgAs from the sera of MS patients [27]–[30] and the specific sites of the neural antigen cleaved by abzymes have been established [31]. Recognition and degradation of MBP peptides by serum auto-Abs was confirmed as a novel biomarker for MS [31]. In MS, anti-MBP abzymes with protease activity can attack MBP of the myelin-proteolipid sheath of axons. The established MS drug Copaxone appears to be a specific inhibitor of MBP-hydrolyzing activity of the abzymes [31]. Consequently, MBP-hydrolyzing abzymes may play an important negative role in MS pathogenesis. At the same time, the similarity in some immunological indexes between MS and SLE can speak in favour of that anti-MBP Abs with proteolytic activity can occur in SLE patients. Recently, it was shown that electrophoretically and immunologically homogeneous IgGs (approximately 86% of SLE patients) purified using several affinity resins including Sepharose with immobilized MBP (MBP-Sepharose) specifically hydrolyze only MBP but not many other tested proteins [9]. Several rigid criteria were applied to show that the MBP-hydrolyzing activity is an intrinsic property of SLE IgGs but not from healthy donors. It was shown, that the immune systems of individual SLE similarly to MS patients can generate a variety of anti-MBP abzymes with different proteolytic properties, which can attack MBP of myelin-proteolipid shell of axons and play an important role in pathogenesis not only MS but also SLE patients.

Anti-MBP abzymes from the sera of MS patients hydrolyze MBP at several sites localized within four known immunodominant regions of MBP [31]. In addition, it was shown that anti-MBP abzymes from the sera of SLE patients hydrolyze MBP at the same four immunodominant sites of MBP [9]. Four peptides corresponding to known immunodominant regions of MBP are encephalytogenic and can play a negative role in the MS and SLE pathogenesis [31].

Taking this into account, it was interesting to study the Ab-dependent hydrolysis of MBP specific sequences in more detail. In this paper, we have analyzed site-specific degradation of two oligopeptides (21- and 25-mer) corresponding to two AGDs of MBP using combination of reverse-phase chromatography (RPhC), thin-layer chromatography (TLC), MALDI spectrometry, affinity chromatography, and enzymic kinetics.

## Results

### Abzyme characterization

In this work, electrophoretically and immunologically homogeneous polyclonal IgGs (pIgGs) were purified from the sera SLE and MS patients as well as healthy donors by sequential chromatography of the serum proteins on Protein G-Sepharose under conditions that remove non-specifically bound proteins, followed by gel filtration under the conditions that destroy immune complexes as in [9]. Electrophoretical and immunological homogeneity of the pIgGs was confirmed respectively by SDS-PAGE with silver staining and by Western blotting similarly to [9], [27]–[30]. To analyze an “average” situation, we have prepared a mixture of equal amounts of homogeneous pIgGs from the sera of ten SLE (sle-IgG_mix_), ten MS (ms-IgG_mix_) patients, and ten healthy donors (hd-IgG_mix_). Then sle-IgG_mix_, ms-IgG_mix_, and hd-IgG_mix_ preparations having affinity for hMBP were separated by affinity chromatography on MBP-Sepharose as in [9]. The fractions of IgG_mix_ eluted from MBP-Sepharose with 3 M NaCl were used in this study. To exclude possible artefacts due to traces of contaminating proteases, these fractions were separated by SDS-PAGE and their proteolytic activity was detected after the extraction of proteins from the excised gel slices as in [9]; only sle-IgG_mix_ and ms-IgG_mix_ preparations were active, when hd-IgG_mix_ was catalytically inactive. The detection of MBP-hydrolyzing activity of these Abs similarly to [9] in the gel region corresponding only to IgGs (150 kDa) together with the absence of any other band of the activity or protein, provided a direct evidence that all pIgG preparations used are not contaminated with canonical proteases. In addition, similarly to [9] it was shown that, in contrast to canonical proteases, the SLE and MS IgG_mix_ purified on MBP-Sepharose specifically hydrolyzed only MBP but not many other tested proteins.

### Ab-dependent hydrolysis of oligopeptides

**Figure 1:**
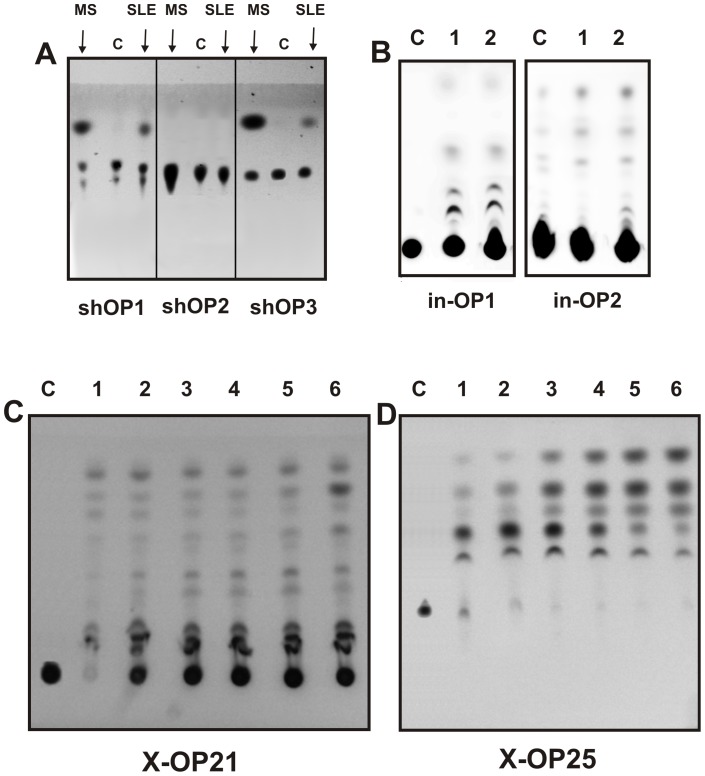
TLC analysis of the hydrolysis of different OPs. A, Boc-Val-Leu-Lys-MCA (shOP1), Pro-Phe-Arg-MCA (shOP2), and Boc-Ile-Glu-Gly-Arg-MCA (shOP3) (5 mM) were incubated for 24 h without Abs (lanesC) and in the presence of 0.05 mg/ml MS IgG_mix_ or SLE IgG_mix_ preparations (lanes shown on the panel) demonstrating comparable relative activities in the hydrolysis of intact MBP. B, Nonspecific in-OP1 and in-OP2 were incubated for 24 h without Abs (lanes 1) and in the presence of 0.05 mg/ml SLE IgG_mix_ (lanes 2); lanes C correspond to in-OP1 and in-OP2 before incubation. C, Specific X-OP21 (C) and X-OP25 oligopeptides were incubated for 7 h with hd-IgG_mix_ (lane C) and in the presence of 0.02 mg/ml sle-IgG_mix_. These OPs were used in different concentrations (mM): 0.05 (lanes 1), 0.1 (lanes 2), 0.2 (lanes 3), 0.3 (lanes 4), 0.4 (lanes 5), and 0.5 (lanes 6).

It was shown previously, that thyroglobulin-directed proteolytic IgGs effectively hydrolyzed not only thyroglobulin but also Pro-Phe-Arg-methylcoumarinamide (MCA) at the Arg-MCA bond with a significantly lower affinity [32]. Polyclonal IgG preparations from patients with rheumatoid arthritis also displayed Pro-Phe-Arg-MCA-hydrolyzing activity [32]. Anti-integrase IgGs and IgMs from HIV-infected patients hydrolyze not only globular HIV-1 integrase but also different specific and nonspecific tri- and tetraoligopeptides [33], [34]. Therefore, first we have analyzed the efficiency of hydrolysis of nonspecific tri- and tetrapeptides using sle-IgG_mix_ and ms-IgG_mix_ purified by affinity chromatography on MBP-Sepharose. It was shown that anti-MBP sle-IgG_mix_ and ms-IgG_mix_ cannot hydrolyse short peptide Pro-Phe-Arg-MCA (sh-OP1), but after 24–48 h of the incubation they cleavage Boc-Val-Leu-Lys-MCA (sh-OP1) and Boc-Ile-Glu-Gly-Arg-MCA (sh-OX3) with very low efficiency (Fig. 1A). The relative rate of the MCA formation was approximately 4-5- (sh-OP2) and 20-25-fold (sh-OX3) higher in the presence of ms-IgG_mix_ than that for sle-IgG_mix_. Similar situation was observed for longer nonspecific 20-mer oligopeptides in-OP1 and in-OP2 (Fig 1B) corresponding to viral integrase, that, as revealed by a MALDI analysis, contain several sites of IN cleavage in the case of anti-IN IgGs from HIV-infected patients [33], [34]. Nonspecific in-OP1 and in-OP2 oligopeptides corresponding to HIV integrase were slightly hydrolyzed nonspecifically after 24 of the incubation, but there was no detectable difference in the fluorescence intensities of the spots after the incubation of these OPs without (lanes 2) and with sle-IgG_mix_ (lanes 3) (Fig. 1B). Consequently, if sle-IgG_mix_ can hydrolyze nonspecific in-OP1 and in-OP2, this hydrolysis is a very negligible.

First, we have shown that all ten individual SLE IgG preparations before purification on MBP-Sepharose produced, according to TLC, the same products of specific (corresponding to MBP) X-OP21 and X-OP25 oligopeptides cleavage, but every preparation was characterized by a specific ratio of formation of different cleavage products. In addition, using TLC it was shown when sle-IgG_mix_ and ms-IgG_mix_ after purification on MBP-Sepharose produce the same X-OP hydrolysis products, but at remarkably different ratios. To analyze in more detail an “average” situation concerning hydrolysis of X-OP21 and X-OP25 by SLE anti-MBP IgGs we have used sle-IgG_mix_; Figs 1C and 1D demonstrate efficient hydrolysis of these specific encephalytogenic OPs by sle-IgG_mix_.

Thus, anti-MBP IgGs from SLE patients can efficiently hydrolyze not only globular molecules of MBP (not many other tested proteins) [9], but also specific OPs corresponding to AGDs of MBP. Interestingly, sle-IgG_mix_-dependent proteolysis of both specific OP21 and OP25 leads to the formation of several products corresponding to 5-10 fluorescent spots (Fig. 1).

**Figure 2:**
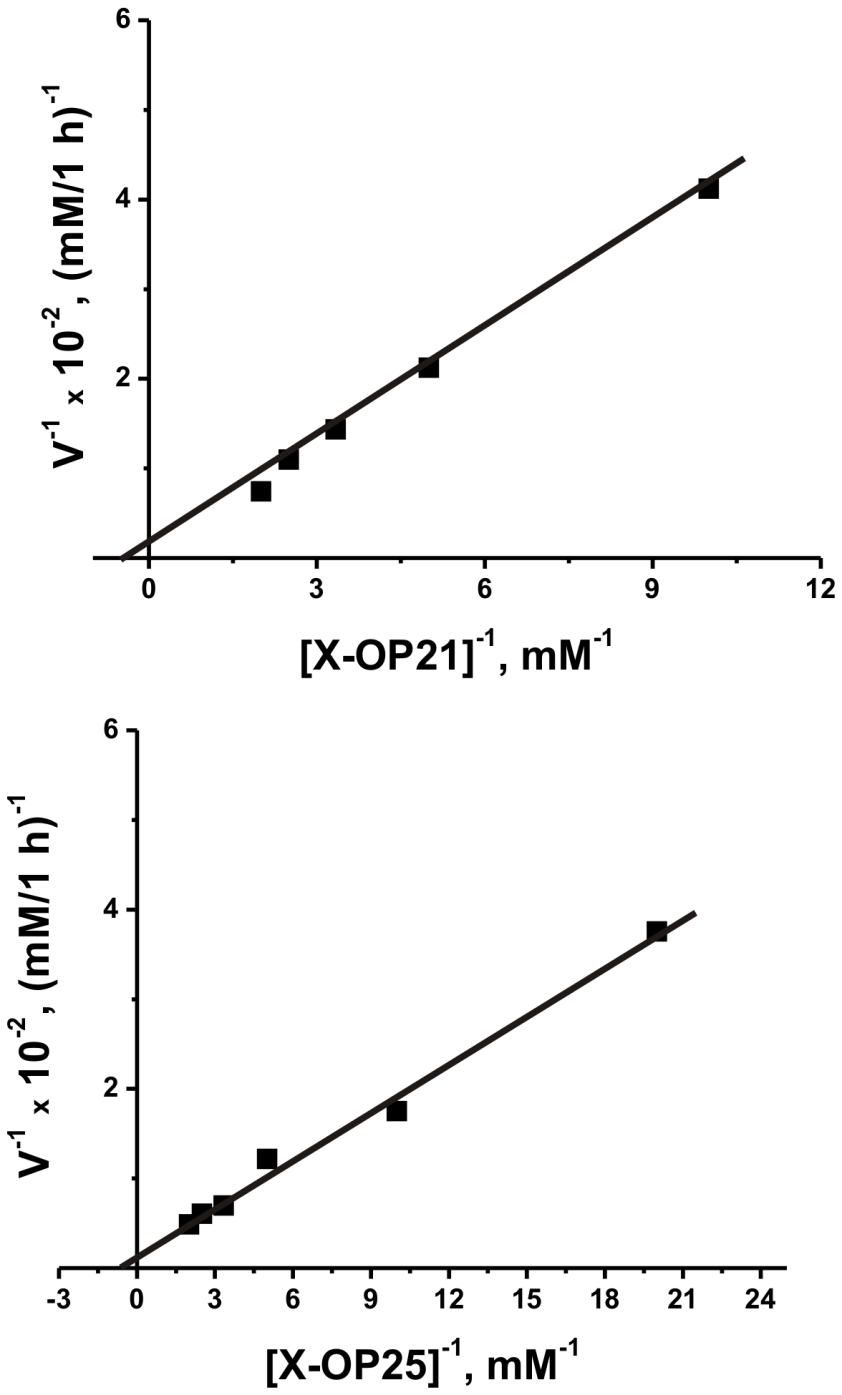
Determination of the *K_m_* and *V*_max_ values for OP21 (A) and OP25 (B) in the reaction catalyzed by sle-IgG_mix_ (0.1 µM) using a Lineweaver–Burk plot. The reactions were performed as described in Materials and Methods. The average error in the initial rate determination from two experiments for each substrate concentration did not exceed 7–10%.

The dependency of the initial rate on the X-OP21 and X-OP25 concentrations in the reaction catalyzed by sle-IgG_mix_ was consistent with Michaelis–Menten kinetics (for example, Fig. 2). The *K_m_* and apparent *k_cat_* values for X-OP21 (2.8±0.3 mM; 1.1±0.1min^−1^) and X-OP25 (1.6±0.2 mM; 1.4±0.2 min^−1^) were estimated.

### MALDI spectrometry analysis of specific peptides hydrolysis

Fig. 1 demonstrates that hydrolysis of specific X-OP21 and X-OP25 by anti-MBP sle-IgG_mix_ produces several fluorescent oligopeptides, the relative amounts of which increase with the increase in the concentration of these OPs. TLC alone cannot unambiguously determine the sequences of these products, since their TLC mobility depends on many factors including the amino acid content, relative hydrophobicity, the nature of the terminal amino acids, etc. To identify major sites of IgG-mediated proteolysis of these OPs, we analyzed products of peptide cleavage by a combination of RPhC, TLC, and MALDI massspectrometry.

**Figure 3:**
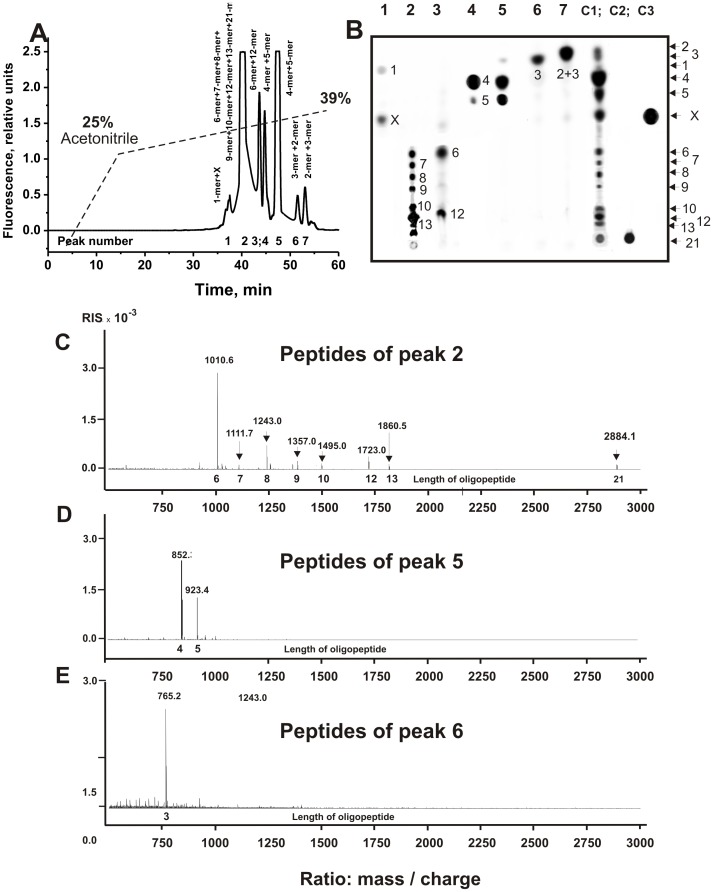
Profile of RPhC of SLE IgG_mix_-dependent products of X-OP21 relatively deep hydrolysis (A) and analysis of X-OP products of the hydrolysis corresponding to different peaks after RPhC by TLC (B) or massspectrometry (C–E): (–), relative fluorescence (A). Numbers of lines in panel B coincide with numbers of peaks on panel A; lanes C1, C2, and C3 correspond to the products of reaction mixture before their separation by RPhC, X-OP25 incubated in the absence of IgGs, and a free fluorescent compound X, respectively. The arrows (and numerals on panel B) indicate the positions of OPs of different length and compound X. Panels C, D, and E demonstrate the MALDI spectrum signals corresponding to the products eluted under RPhC in peaks 2, 5 and 6, respectively. See Materials and Methods for other details.

First, we have analyzed the products of nearly complete X-OP21 hydrolysis after 7 h of incubation (Fig. 3). Seven major and several very small peaks corresponding to fluorescent products of X-OP21 hydrolysis were revealed by RPhC (Fig. 3A). The products of all peaks were analyzed by TLC (Fig. 3B) and by massspectrometry (Figs 3C and 3D). One can see that only the 4^th^ and 7^th^ RPhC peaks according to TLC contain a single predominant product of the hydrolysis. According to TLC and massspectrometry major peak 2 contains seven products of the cleavage (n = 6, 7, 8, 9, 10, 12, and 13) and initial non-cleaved X-OP21 having comparable affinity to RPhC-resin (Fig. 3A), but different mobility at TLC (Fig. 3B). Badly separated 4^th^ and 5^th^ peaks (Fig. 3A) contained mainly 4- and 5-mers (Fig. 3D), while 7^th^ peak 2- and 3-mer X-OPs.

**Figure 4:**
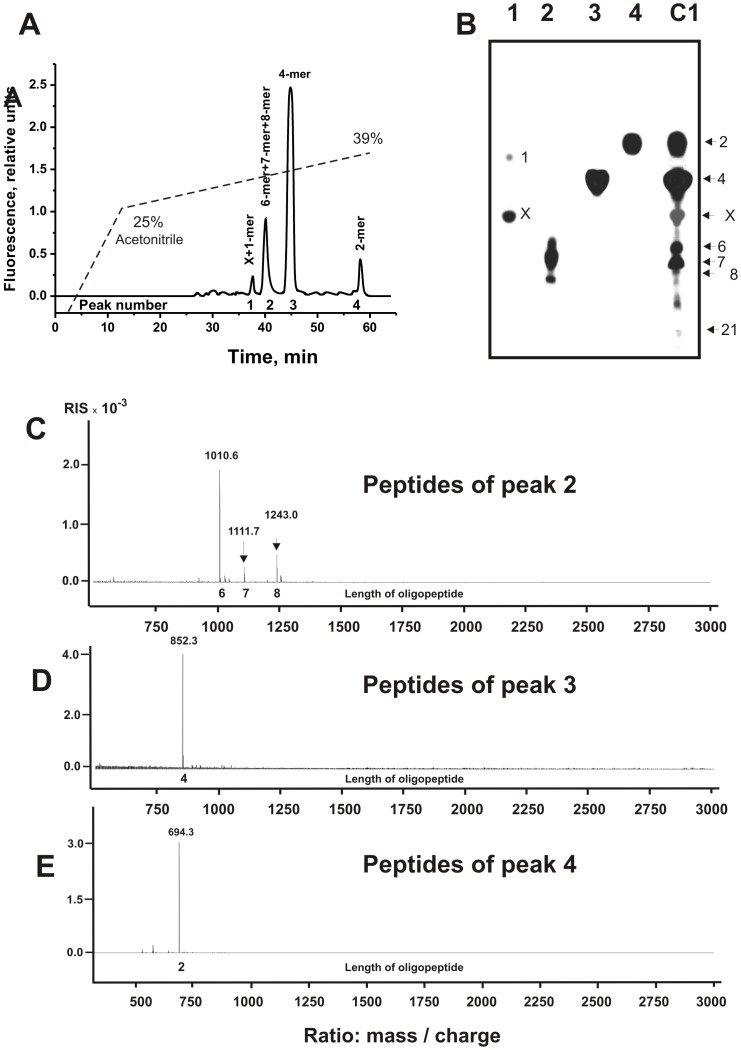
Profile of RPhC of sle-IgG_mix_-dependent products of a complete hydrolysis of X-OP21 (A) and analysis of the products corresponding to different peaks after RPhC by TLC (B) or massspectrometry (C–E): (–), relative fluorescence (A). Numbers of lines in panel B coincide with numbers of peaks on panel A; lane C1 corresponds to the products of reaction mixture before their separation by RPhC. The arrows (and numerals on panel B) indicate the positions of OPs of different length and compound X. Panels C, D, and E demonstrate the MALDI spectra corresponding to the products of the hydrolysis eluted under RPhC in peaks 2, 3, and 4, respectively (Fig. 4A). See Materials and Methods for other details.

Fig. 4A demonstrates the data of RPhC of the cleavage products corresponding to X-OP21 after its complete hydrolysis (12 h). In this case there was observed only four major and many very small peaks. According to TLC (Fig. 4B, lane 2) and massspectrometry (Fig. 4C), peak 2 contained mainly three products: 6-, 7-, and 8-mer X-OPs. Peak 3 corresponded to 4-mer (Fig. 4D), while 4^th^ peak to 2-mer X-OP (Fig. 4E). Thus, long products of the hydrolysis finally can be hydrolyzed by SLE-Abs to short X-dimer and X-tetramer. In addition, after a long incubation small amount of a free X-fluorescent compound and X-monomer were revealed (peak 1, Figs 4A and 4B) using TLC and massspectrometry. It should be mentioned that massspectrometry analysis of products of X-OP21 hydrolysis after 3 h of incubation has shown that three OPs with low TLC mobility (Fig. 1C) correspond to 10-, 12-, and 13-mer X-OPs.

**Figure 5:**
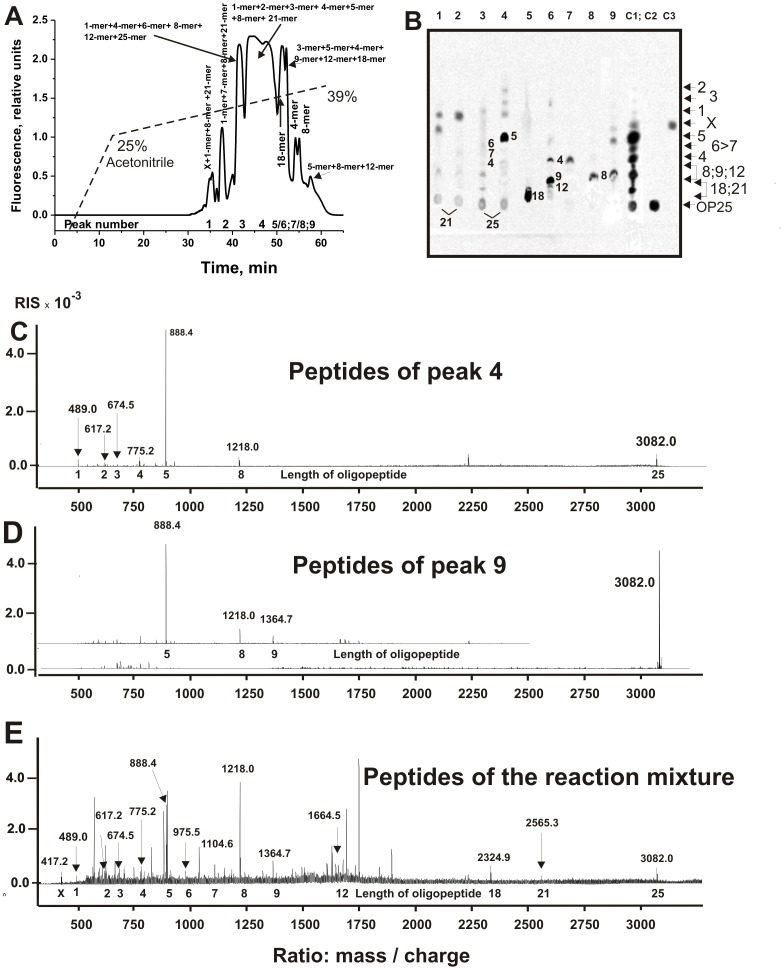
Profile of RPhC of sle-IgG_mix_-dependent products of X-OP25 relatively deep hydrolysis (A) and analysis of the products of the hydrolysis corresponding to different peaks after RPhC by TLC (B) or massspectrometry (C–E): (–), relative fluorescence (A). Numbers of lines in panel **B** coincide with numbers of peaks on panel **A**; lanes C1, C2 and C3 correspond to the products of reaction mixture before their separation by RPhC, X-OP25 incubated in the absence of IgGs, and a free fluorescent compound X, respectively. The arrows (and numerals on panel **B**) indicate the positions of OPs of different length and compound X. Panel **C** demonstrates the MALDI spectrum of the products corresponding to peak 4, while panel D to peak 9 (Fig. 5A) and the intact X-OP25 before its hydrolysis, respectively. MALDI spectrum of all products of X-OP25 hydrolysis after 24 h of its incubation corresponding to non-fractionated reaction mixture is given on panel **E**. The length of the X-OPs is given on the bottom. See Materials and Methods for other details.

Nine brightly expressed and several additional very small peaks corresponding to fluorescent products of X-OP25 medium hydrolysis were revealed by RPhC (Fig. 5A). After RPhC the same products of the hydrolysis were identified by TLC and by massspectrometry in several peaks (Fig. 5). It means that some individual products of X-OP25 hydrolysis can be eluted from the sorbent by different concentrations of acetonitrile. The reaction mixture contains Tris-HCl and trifluoroacetic acid, which components can interact with positively and negatively charged amino acid residues of products of the X-OP25 hydrolysis. In addition, one cannot exclude that some of OPs can form interpeptide complexes. An existence of multiple forms of the X-OP products can lead to the elution of these forms of the same X-OPs from the sorbent by different concentration of acetonitrile.

Using the combination of RPhC, TLC, and MALDI-TOF analysis of the products of the X-OP25 hydrolysis were identified. For example, the highest peak 4 according to TLC (Fig 5B, lane 4) and massspectrometry (Fig. 5C) contained several X-OPs: 5-mer>>25-mer>3-mer≥2-mer≥1-mer≥4-mer≥8-mer. PanelD demonstrates the spectrum of initial X-OP25 and the signals of peptides corresponding to major OPs of peak 9. The signals of all products of X-OP25 hydrolysis before their separation by RPhC are given on panel E. It was shown that at the beginning of the reaction (1-3 h of the incubation) 18-mer OP is a major product of the X-OP25 cleavage, while the formation of long products containing 8, 9, 12, and 21 amino acid residues occurs with significantly lower rate.

As it was shown above, the relative content of different length X-OPs in the final reaction mixtures depend upon concentrations of X-OP21, X-OP25 (Fig. 1), and IgGs as well as time of the incubation. An approximate relative content of different products of in the final reaction mixtures was estimated taking into account a relative total fluorescence of the spots with different mobility after TLC (Figs 3B and 5B). The data for X-OP21 is given in Table 1. Since several products of different length in the case of X-OP25 demonstrated comparable mobility and were badly separated (Fig. 5B), their relative content was estimated approximately as a sum of several products (Table 2).

**Table 1:** The data of RPhC, TLC, and MALDI analysis of molecular masses of fluorescent oligopeptides forming after incubation of X-OP21 with sle-IgG_mix._

Num-ber of AA	Sites of cleavage of X-OP21 (OPs found by massspectrometry in the reaction mixture and peaks after RPhC)	Mol. mass, Da (ratio mass/charge, H^+^-form)		Peak number after RPhC (Fig. 3A)	Lane number after TLC, (Fig. 3B)	Relative content at a incomplete hydrolysis of OP,%§
		Calculated	Experimental			
0	X*	417	416.75	1;C1**	1***	8-11 **
1	X- Y	58.94	581.0	1;C1	1	≤1.0
2	X- YL	694.1	694.3	6(7);C1	7(6)	1–2
3	X- YLA	765.17	765.2	6(7);C1	6(7)	2–3
4	X- YLAS	852.25	852.4	4(5);C1	4(5)	33–37
5	X- YLASA	923.33	923.4	5(4);C1	5(4)	11–15
6	X- YLASAS	1011.5	1010.6	2(3);C1	2(3)	3–6
7	X- YLASAST	1111.51	1112.7	2(4);C1	2	2–3
8	X- YLASASTM	1242.71	1243.0	2;C1	2	5–6
9	X- YLASASTMD	1356.79	1357.0	2;C1	2	1–2
10	X- YLASASTMDH	1494.43	1495.0	2;C1	2	3–4
12	X- YLASASTMDHAR	1722.7	1723.0	2(3);C1	2(3)	7–11
13	X- YLASASTMDHARH	1860.34	1860.5	2;C1	2	3–4
21	YLASASTMDHARHGFLPRRHR	2884.07	2884.1	2;C1	2	4–5

* Free fluorescent compound; all analyzed OPs contained fluorescent X-component.

** The same products of the hydrolysis separated by RPhC (Fig. 3A) were revealed in several peaks by MALDI spectrometry; the main peaks are marked in bold, while additional peaks containing low amount of the same OPs are shown in parentheses. C1 reflects the presence of signal corresponding to the analyzed product in spectrum of total reaction mixture.

*** The same several products of the hydrolysis corresponding to each peak after RPhC (Fig. 3A) were revealed not only by MALDI spectrometry, but also by TLC (Fig. 3B).

§ The relative content of different products after incomplete hydrolysis of X-OP21 was performed taking into account the data of TLC analysis (Fig. 3B); a range of the values from three repeats is given.

**Table 2:** The data of RPhC, TLC, and MALDI analysis of molecular masses of fluorescent ligopeptides forming after incubation of X-OP25 with sle-IgG_mix_.

Num-ber of AA	Sites of cleavage of X-OP25 (OPs found by massspectrometry in the reaction mixture and peaks after RPhC)	Mol. mass, Da (ratio mass/charge, H^+^-form)		Peak number after RPhC (Fig. 5A)	Lane number after TLC, (Fig. 5B)	Relative content of different products after a medium hydrolysis of X-OP25,%^§^
		Calculated	Experimental			
0	X*	417.0	417.2	1;C1**	1***	≤1
1	X- A	488.84	489.0	1;2;C1	1;2(3)	≤1
2	X- AQ	616.97	617.2	4;C1	4	≤1
3	X- AQG	674.02	674.5	4 (6);C1	4(6)	≤1
4	X- AQGT	775.13	775.2	6;7(3.4) C1	6;7(3;4)	16–20
5	X- AQGTL	888.29	888.4	4(9);C1	4(9)	32–36
6	X-AQGTLS	975.36	975.5	2;3;C1	2;3	Together 6–7; 6>>7
7	X-AQGTLSK	1104.54	1104.6	2; C1	2	
8	X-AQGTLSKI	1217.7	1218.0	8;9(1;2;3); C1	8;9(1;2;3)	Together 13–17
9	X-AQGTLSKIF	1364.87	1364.9	6 (9);C1	6 (9)	
12	X-AQGTLSKIFKLG	1664.26	1664.5	3;6;C1	3;6	
18	X-AQGTLSKIFKLGGRDSRS	2323.94	2324.9	5(6);C1	5(6)	16–19
21	X-AQGTLSKIFKLGGRD SRSGSP	2565.18	2565.3	1;2; C1	1;2	Together 10–15
25	X-AQGTLSKIFKLGGRDS RSGSPMARR	3081.84	3082.0	3;4;C1	3;4	

* Free fluorescent compound; all analyzed OPs contained fluorescent X-component.

** The same products of the hydrolysis separated by RPhC (Fig. 5A) were revealed in several peaks by MALDI spectrometry; the main peaks are marked in bold, while additional peaks are shown in parentheses. C1 reflects the presence of signal corresponding to the analyzed product in the spectrum of total reaction mixture.

*** The same several products of the hydrolysis corresponding to each peak after RPhC (Fig. 5A) were revealed not only by MALDI spectrometry, but also by TLC (Fig. 5B)

## Discussion

Autoantibody-mediated tissue destruction is among the main features of organ-specific autoimmunity. It was recently shown that, anti-MBP IgGs from the sera of patients with MS [27]–[31] and SLE [9] efficiently hydrolyze globular MBP but not many other tested proteins. No activity was found for IgG fraction of healthy donors in the hydrolysis of MBP or different OPs (Fig. 1) [27]–[31]. The sites of MBP cleavage by MS IgGs determined by mass spectrometry were localized within four known immunodominant regions of MBP [31]. SLE IgGs hydrolyzed MBP within the same immunodominant regions of MBP [9]. It should be mentioned that for the identification of these sites only the largest peptides generated by Ab-dependent hydrolysis of intact MBP after short times of the incubation were used [31]. At the same time, we have seen that long incubation of MBP with MS and SLE IgGs (48–72 h), especially with abzymes possessing high proteolytic activity, led to the formation of short and very short fragments. Similar situation was observed for anti-IN abzymes from HIV-infected patients in the hydrolysis of viral integrase [33], [34]. It means that the total pools of various IgGs can contain different subfractions of anti-protein abzymes, which are capable to hydrolyze a target protein in many sites but with different rates. Theoretically, a mammalian immune system can produce up to 10^6^ variants of Abs against a single antigen including abzymes and various Abzs can be different in their specificity toward their substrates. To understand possible general features of production and mechanisms of the action of different monoclonal abzymes in the total IgG pool leading finally to the formation of short OPs only, it was interesting and useful to analyze not only the major cleavage sites but also various minor ones.

**Figure 6:**
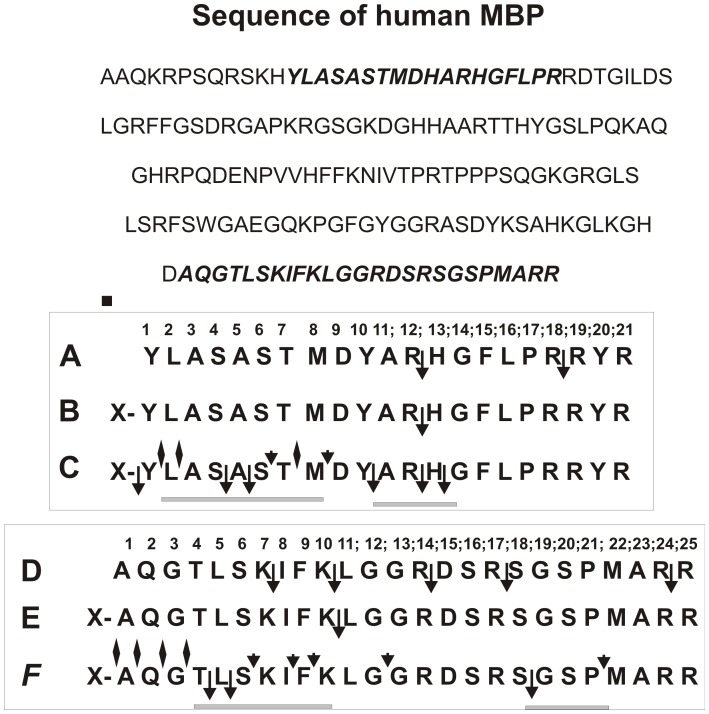
Complete sequence of human MBP (on the top) and all sites of cleavage of X-OP21 (C) and X-OP25 (F) determined using a combination of RPhC, TLC, and massspectrometry of detectable major and minor products of these OPs hydrolysis by sle-IgG_mix_. The positions of OP21 and OP25 sequences in the human MBP sequence are shown in bold. Panels **A** and **D** show all possible sites of these OPs cleavage by trypsin, while panels **B** and **E** demonstrate the major cleavage sites of MBP, which were found previously in the case of hydrolysis of globular intact MBP by MS IgGs [31]. All sites corresponding to major and moderate products of the cleavage are shown by long and short arrows respectively, while to minor ones by diamonds (panels **C** and **F**).

In the case of MS IgGs, the major sites of Ab-dependent cleavage of globular MBP within the sequences corresponding to OP21 and OP25 were identified earlier [31]; they are shown on Fig. 6B and 6E. Interestingly, one cleavage site of OP21 coincides with one of two possible trypsin-dependent sites of this OP hydrolysis (Fig. 6A). In the case of OP25 there are five potential sites of its hydrolysis by trypsin (Fig. D), but globular MBP was digested by MS IgGs only at one of these sites (Fig. 6E) [31].

In this article for a more exact identification of the major and minor sites of the MBP cleavage by SLE IgGs we have used several nonspecific and two specific OPs corresponding to two cleavage sequences of MBP (X-OP21 and X-OP25; Fig. 6). It was shown, that anti-MBP sle-IgG_mix_ hydrolyze efficiently only specific X-OP21 and X-OP25, while cannot efficiently hydrolyze different nonspecific short and long OPs. To identify major sites of abzyme-dependent proteolysis of X-OP21 and X-OP25, we analyzed these peptide cleavage products by a combination of RPhC, TLC, and MALDI spectrometry. Using conditions of a deep Ab-dependent degradation of these OPs several major and minor cleavage sites were identified (Figs 6C and 6F).

As one can see from Fig. 1, the relative amounts of different products of X-OP21 and X-OP25 cleavage significantly depend upon the concentration of these OPs. We have estimated the relative amount of the products after a medium (for example, Fig. 1C) and deep hydrolysis of OPs hydrolysis (Figs 3 and 5; Tables 1 and 2). It should be mentioned, that at the beginning of the X-OP21 cleavage reaction (1-3 h of the incubation) three fluorescent spots corresponding to three OPs (X-OP12>XOP13≥X-OP10) were revealed (Fig. 1C). Overall, in the case of hydrolysis of X-OP21 five major sites of cleavage corresponding to 4-, 5-, 10-, 12-, and 13-mer X-OPs were identified (Fig. 6C). In addition, a remarkable accumulation of the free fluorescent X-compound was observed. The rate of X-OP21 cleavage leading to a formation of 6-mer and 8-mer X-OPs was significantly lower. The relative content of 1-, 2-, 3-, 7-, and 9-mer X-OPs did not exceed 1–3% (Table 1).

^§^The relative content of different products after a medium hydrolysis of X-OP25 was performed taking into account the data of TLC analysis (Fig. 5B); a range of the values from three repeats is given.

Three major sites of cleavage (10-, 12-, and 13-mer X-OPs) are localized in one cluster. One of them (12 -mer OP, cleavage of R-H bond) coincides with the cleavage site identified recently for 21-mer sequence of intact MBP (it coincides with trypsin-dependent site) in the case of IgGs from MS patients (Fig. 6B; [31]), while two other were new sites. After short time of the incubation in parallel with the formation of long OPs several shorter X-OPs was observed (Fig. 1C). Deeper incomplete hydrolysis of X-OP21 (7 h) results in the degradation of long 10-18-mer X-OPs and the formation of several additional products of the cleavage including major OPs: 4-mer>5-mer>8-mer (Fig. 3B, Table 1). After practically complete hydrolysis of the initial X-OP21 the reaction mixture contained only six products of the cleavage: 4-mer>2-mer>7-mer>6 mer≥8-mer X-OPs (Fig. 4B). Taken together, one can say that cleavage sites corresponding to the formation of 13-mer, 10-mer, 5-mer and especially 12-mer and 4-mer X-OPs may be considered as major sites of the cleavage. Several sites correspond to a remarkably lower efficiency of the hydrolysis (6- and 8-mer OPs), and finally there are several sites of very low rate of the hydrolysis (1-, 2, 3,-7,-, 9-mer X-OPs). All sites of IgG-dependent hydrolysis of X-OP21 are shown on Fig. 6C. Interestingly, eight amino acid residues of YLASASTM sequence correspond to a specific cluster of seven sites of very weak, medium and strong cleavage (Fig. 6C).

Similar situation was observed for X-OP25. After 3 h of the hydrolysis of X-OP25 a fast formation of 5-mer, 4-mer, and 18-mer OPs was observed. After a deep hydrolysis the same 5-, 4-, and 18-mer OPs were major products (Fig. 5B, Table 2). Taken together, only three sites of X-OP25 may be considered as major (5-, 4-, and 18-mer OPs), while other sites correspond to a moderate of a low efficiency of the X-OP25 digestion (Fig 6F). Only one cleavage site of intact globular MBP in the case of MS IgGs was determined and it corresponds to the formation of 10-mer OPs and coincides with trypsin-dependent site (Fig. 6E). However, we did not find X-OP10 among products of X-OP25 cleavage by SLE anti-MBP IgGs; a remarkable digestion was observed at three neighboring sites (8-, 9- and 12-mer OPs) (Fig. 6F, Table 2). The 10-mer sequence AQGTLSKIFK contains eight clustered sites of the cleavage (Fig. 6D).

The first question is why in the case of SLE anti-MBP IgGs there are more major cleavage sites of sequences corresponding to X-OP21 and X-OP25 than those for MS abzymes in the case of intact MBP as substrate. It was recently shown that incubation of many individual IgGs with iodoacetamide (a specific inhibitor of thiol proteases) or pepstatin A (a specific inhibitor of acidic proteases) has moderate effect (5-15%) on SLE Ab-dependent hydrolysis of MBP [9]; the same was demonstrated for MS IgGs, IgAs and IgMs [27]-[31]. However, PMSF, AEBSF (specific inhibitors of serine proteases), and EDTA (an inhibitor of metalloproteases) significantly suppressed the proteolytic activity of SLE and MS pIgGs in the case of MBP and OPs as substrates [9]. In contrast to MS IgGs, abzymes from SLE patients are more sensitive to EDTA like canonical metalloproteases and less sensitive to specific inhibitors of serine-like proteases [9]. The cleavage sites of Me^2+^- dependent fractions of the total pool of SLE anti-MBP abzymes may be different from those of serine-like proteolytic IgG fraction dominating in the sera of MS patients.

Then, for the revealing of the SLE Ab-dependent cleavage sites we have used X-OPs, while the cleavage sites in the case of MS IgGs were identified using digestion of intact globular MBP [31]. In this connection it should be mentioned, that catalytic centers of proteolytic abzymes (including MS and SLE anti-MBP IgGs [9], [27]) are usually located on the light chain, while the heavy chain is more often responsible for specific antigen recognition and increased antigen affinity for Abs [1]-[8]. Intact proteins usually interact with both light and heavy chains of abzymes, thus ensuring the specificity of the target protein recognition and its cleavage. Specific binding of globular protein with the heavy chains of abzymes can determine what fragment of protein sequences may be localized in the active centers of the light chains. At the same time, short oligopeptides may interact mostly with the light chain, which possesses a lower affinity for substrates. For example, the affinity of SLE pIgGs for hMBP (*K_m_* = 0.6–2.7 µM [9]) is comparable with that for MS IgGs (*K_m_* = 0.9–5.0 µM [30]) and corresponds to the typical affinity of abzymes for different protein antigens (*K_m_* = 0.038–30 µM) [1]–[8]. At the same time, the affinity of SLE anti-MBP IgGs for X-OP21 and X-OP25 (*K_m_* = 1.6–2.8 mM; Fig. 2) approximately three orders of magnitude lower that to MBP. Therefore depending on the sequence, IgG-dependent hydrolysis of OPs may in principle be less specific or completely nonspecific in comparison with intact globular MBP.

The second question is whether the minor sites of OPs cleavage have any biological significance. In this connection it should be mentioned that sle-IgG_mix_ cannot effectively hydrolyze short nonspecific tri- or tetrapeptides and long OPs (Fig. 1). In addition, between products of the hydrolysis of X-OP21 and X-OP25 there is only a limited number of shorter X-OPs, which do not cover all possible sites of these OPs digestion. Notably, light chains of IgGs from patients with different diseases can hydrolyze not only intact specific protein substrates, but also different tri- and tetrapeptides demonstrating significantly lower affinities; the *K_m_* for Pro-Phe-Arg-MCA were determined in the case of IgGs from Hashimoto thyroiditis (18 µM, *k_cat_* = 6.3×10^−2^ min^−1^ [35]). The affinity (*K_m_*) of these very short OPs for intact IgGs and for monoclonal light chains corresponding to Abs against different proteins are to some extent comparable: anti-VIP (0.012 mM, *k_cat_* = 6.8×10^−3^ min^−1^ [36]), anti-prothrombin abzymes (0.1 mM, *k_cat_* = 2.6×10^−2^ min^−1^ [37]), proteolytic Bence Jones proteins (different MCA-peptides; 0.015–0.29 mM, *k_cat_* = (2.1–9)×10^−2^ min^−1^ [38]). These data support the hypothesis that all short peptides interact mainly with light chains of intact Abs.

It was shown that anti-integrase IgGs from HIV-infected patients efficiently hydrolyze specific 17-20-mer peptides corresponding to AGDs of viral integrase, nonspecific tri- and tetrapeptides, and 17-25-mer nonspecific OPs of human myelin basic protein AGDs, suggesting poor discriminatory properties of the sites for substrate binding and hydrolysis located on the light chains of anti-IN IgGs from HIV-infected patients [33]. Taken together, all data support the hypothesis that short peptides can interact directly with the light chains of intact Abs.

Protein-binding sites of light chains of SLE anti-MBP IgGs are much more selective, but these abzymes also can with very low efficiency hydrolyzed short nonspecific MCA-peptides. Therefore, one cannot exclude that the observed multiple sites of the hydrolysis of specific OPs at some sites may be to some extent nonspecific. However, using MALDI mass spectrometry we have recently identified 40sites of integrase cleavage by anti-IN IgGs from HIV infected patients [33], which are localized within seven known immunodominant regions of IN. Interestingly, all these cleavage sites were clustered within the AGDs. For example, three clusters of cleavage sites were located within the long AGD3. A block of 12 closely spaced cleavage sites corresponded to the N-terminal part of AGD4. Similar situation was observed in the case of X-OP21 and X-OP25 degradation by SLE IgGs; several sites of the major and minor cleavage are clustered (Fig. 6).

On one hand, it cannot be excluded that the digestion of the X-OP21 and X-OP25 at different sites is catalyzed by different monoclonal Abzs present in the total pool of polyclonal anti-MBP abzymes. Depending on their affinity, specificity, and relative activity, different monoclonal abzymes may participate at different stages of MBP degradation; faster cleavage may lead to the formation of several major products, while significantly slower cleavage, to the minor ones. However, it is possible that single abzymes can cleave MBP at multiple sites in a sequential series of cleavage reactions. For example, several different monoclonal light chains (corresponding to SLE) obtained using phage display specifically hydrolyze only myelin basic protein, but are very different in their site specificity (A. M. Bezuglova and G. A. Nevinsky, personal communication). Some monoclonal light chains efficiently hydrolyze only OPs corresponding to one of four known AGDs of MBP. However, we have found monoclonal light chain preparations hydrolyzing OPs corresponding to two, three and even four AGDs of MBP with comparable rates. The deep hydrolysis of intact MBP led to the formation of many short peptides. Thus, one cannot exclude that monoclonal anti-MBP abzymes in the total Ab pool can also be significantly different in their site specificity. Altogether, it seems reasonable to suggest that the observed multiplicity of the specific OPs cleavage sites may be explained both by differences in the site specificity of many monoclonal abzymes and by the existence of single abzymes possessing low site specificity in the MBP hydrolysis.

In addition, the sera of MS and SLE patients are characterized by increased concentration of free light chains of IgGs [39]. Therefore, one can not exclude that free light chains can also be important for the hydrolysis of MBP at minor sites of cleavage.

Taken together, we have shown for the first time an enormous multiplicity of MBP cleavage sites for abzymes from patients with SLE.

## Materials and Methods

### Chemicals, donors, and patients

Human MBP was from the Department of Biotechnology, Research Center of Molecular Diagnostics and Therapy (Moscow); all other chemicals including Protein G-Sepharose were from Sigma or Pharmacia. MBP-Sepharose was prepared by immobilizing human MBP on BrCN-activated Sepharose according to the standard manufacturer's protocol. Sera of 14 patients (27–60 yr old; men and women) with clinically definite SLE were used to study proteolytic abzymes. The SLE diagnosis was confirmed and its reliability was checked according to the criteria developed by the American Rheumatoid Association. For comparison we have used the sera of 10 healthy donors and of 12 patients (16–55 yr old; men and women) with clinically definite MS according to the Poser criteria [16]. The blood sampling protocol conformed to the local human ethics committee guidelines (Ethics committee of Novosibirsk State Medical University, Novosibirsk, Russia; Institutional ethics committee specifically approved this study) including written consent of patients and healthy donors to present of their blood for scientific purposes in accordance with Helsinki ethics committee guidelines.

### Antibody purification and analysis

Electrophoretically and immunologically homogeneous pIgGs from healthy volunteers, SLE, and MS patients were prepared by sequential affinity chromatography of the serum proteins on protein G-Sepharose and FPLC gel filtration on a Superdex 200 HR 10/30 column as in [9]. SDS-PAGE analysis of Abs for homogeneity was performed in 12% or 4–15% gradient gels (0.1% SDS); the polypeptides were visualized by silver and Coomassie R250 staining [9].

We have prepared three mixtures of equal amounts of homogeneous IgGs from the sera of ten SLE (sle-IgG_mix_), ten MS (ms-IgG_mix_) patients, and ten healthy donors (hd-IgG_mix_). The sle-IgG_mix_, ms-IgG_mix_, and hd-IgG_mix_ preparations were chromatographed on Sepharose bearing immobilized MBP similarly to [9]. The column (3 ml; 0.7 mg MBP per ml of Sepharose) was equilibrated with 50 mM Tris-HCl (pH 7.5) containing 50 mM NaCl; the protein was applied, and the column was washed with the same buffer to zero optical density. IgGs were eluted from MBP-Sepharose with the same buffer containing different concentrations of NaCl (0.1–3 M) and then with 2–3 M MgCl_2_. Fractions after chromatography were dialyzed against 50 mM Tris-HCl (pH 7.5) containing 50 mM NaCl and concentrated using Amicon-50. All 5 major fractions of sle-IgG_mix_ and ms-IgG_mix_ eluted from MBP-Sepharose with different concentrations of NaCl (0.1–3.0 M) and MgCl_2_ (2–3 M) were active, while fractions of hd-IgG_mix_ preparation were inactive in the hydrolysis of MBP. In order to protect the Ab preparations from bacterial contamination IgGs after all stages of purification were sterilized by filtration through a Millex filter (pore size 0.2 µm). In this study we have used sle-IgG_mix_ and ms-IgG_mix_ fractions eluted from MBP-Sepharose with 3 M NaCl demonstrating comparable activities in the hydrolysis of intact MBP. These fractions of Abs were marked in the following text as sle-IgG_mix_ and ms-IgG_mix_.

### Ab proteolytic activity assay

The reaction mixture (10–100 µl) for analysis of OP-hydrolyzing activity of IgGs containing 20 mM Tris-HCl (pH 7.5), 3 mM NaCl, 0.05–1.0 mM one of two specific or various nonspecific OPs, and 0.001–0.02 mg/ml of IgGs (purified on BMP-Sepharose), was incubated for 1–24 h at 30°C. Specific X-OP21 (X-YLASASTMDHARHGFLPRRHR) and X-OP25 (X-AQGTLSKIFKLGGRDSRSGSPMARR) corresponding to two of four known IgG-dependent specific cleavage sites of hMBP [31] were used. In the case of X-OP21 only 18 N-terminal residues correspond to the antigenic determinant; this OP contains three additional C-terminal amino acid residues. As controls, we have used OPs corresponding to specific cleavage sites of two known AGDs of HIV-1 integrase, which were identified in the case of abzymes from HIV-infected patients: 20-mer X-EHEKYHSNWRAMASDFNLPP (in-OP1) and 20-mer X-VESMNKELKKIIGQVRDQAE (in-OP2) [33], [34]. All mentioned OPs contained fluorescent residue 6-O-(Carboxymethyl)fluorescein ethyl ester (X) on its N-terminus. In addition, three short fluorogenic peptidyl-4-methylcoumaryl-7-amides (MCA) were used as nonspecific controls: Boc-Val-Leu-Lys-MCA (shOP1), Pro-Phe-Arg-MCA (shOP2), Boc-Ile-Glu-Gly-Arg-MCA (shOP3).

The cleavage products of different OPs were separated by TLC on Kieselgel F60 plates using acetic acid – *n*-butanol – H_2_O (1:4:5) system. The plates were dried and photographed. To quantify the intensities of the fluorescent spots after TLC, X-OP21, X-OP25, and other OPs incubated without IgGs (or with hd-IgG_mix_) was used as controls. Photographs of the plates were imaged by scanning and quantified using GelPro v3.1 software.

In some experiments the cleavage products of specific X-OP21 and X-OP25 were first separated by reverse-phase chromatography on Nucleosil C-18 column (4.6×250 mm) using 0.05% trifluoroacetic acid and gradient of acetonitrile concentration (0 - 80%). The relative amount of various cleavage products was calculated by the fluorescence. Excitation was performed at 320 nm and fluorescence emission detected at 490 nm. The fractions corresponding to different peaks were collected, evaporated to minimal volume and products of the hydrolysis were analyzed by TLC (see above) and by MALDI spectrometry (see below).

### MALDI-TOF analysis Ab-dependent hydrolysis of OPs

In all cases the products of OP hydrolysis were analysed by MALDI-TOF mass spectrometry using a ReflexIII system (Bruker, Germany) equipped with a 337-nm nitrogen laser (VSL-337ND, Laser Science, Newton, MA), 3 ns pulse duration. Saturated solution of cyano-4-hydroxycinnamic acid in a mixture of 0.1% acetonitrile and trifluoroacetic acid (1:2) was used as the matrix. To 1 µl of the reaction mixture containing hydrolyzed OPs before or after their separation by RPhC or TLC, 1 µl of 0.2% trifluoroacetic acid and 2 µl of the matrix were added, and 1 µl of the final mixture was spotted on the MALDI plate, air-dried, and used for the analysis. Calibration of the MALDI spectra was performed using the protein and OP standards I and II (Bruker Daltonic, Germany) in the external and internal calibration mode.

### Determination of the kinetic parameters

The reaction mixtures contained the standard components and different concentrations of OPs. All measurements (initial rates) were taken under the conditions of the pseudo-first order of the reaction within the linear regions of the time courses(<40% of OP hydrolysis). The cleavage products were analyzed by TLC. The activity of IgG_mix_ was determined as a decrease in the percentage of initial X-OPs converted to shorter X-OPs, corrected for the distribution of the fluorescence label between these spots in the control (incubation of X-OPs in the absence of Abs) and taking into account the concentration of each OP in every reaction mixture. The *K_M_* and *V*_max_ (apparent *k_cat_* = V_max_/[Abs]) values were calculated from the dependencies of V versus [OP] by least-squares non-linear fitting using Microcal Origin v5.0 software and presented as linear transformations using a Lineweaver–Burk plot [40]. Errors in the values were within 10–15%. The results are reported as mean±S.E. of at least three independent experiments.
